# Acoustic Identification of Individuals within Large Avian Populations: A Case Study of the Brownish-Flanked Bush Warbler, South-Central China

**DOI:** 10.1371/journal.pone.0042528

**Published:** 2012-08-06

**Authors:** Canwei Xia, Xuanlong Lin, Wei Liu, Huw Lloyd, Yanyun Zhang

**Affiliations:** 1 Ministry of Education Key Laboratory for Biodiversity and Ecological Engineering, College of Life Sciences, Beijing Normal University, Beijing, China; 2 State Key Laboratory of Systematic and Evolutionary Botany, Institute of Botany, Chinese Academy of Sciences, Beijing, China; 3 Division of Biology and Conservation Ecology, School of Science and the Environment, Manchester Metropolitan University, John Dalton East, Manchester, United Kingdom; Utrecht University, The Netherlands

## Abstract

Acoustic identification is increasingly being used as a non-invasive method for identifying individuals within avian populations. However, most previous studies have utilized small samples of individuals (<30). The feasibility of using acoustic identification of individuals in larger avian populations has never been seriously tested. In this paper, we assess the feasibility of using distinct acoustic signals to identify individuals in a large avian population (139 colour-banded individuals) of Brownish-flanked Bush Warbler (*Cettia fortipes*) in the Dongzhai National Nature Reserve, south-central China. Most spectro-temporal variables we measured show greater variation among individuals than within individual. Although there was slight decline in the correct rate of individual identification with increasing sample sizes, the total mean correct rate yielded by discriminant function analysis was satisfactory, with more than 98% of songs correctly recognized to the corresponding individuals. We also found that using a part of randomly selected measured variables was sufficient to obtain a high correct rate of individual identification. We believe that our work will increase confidence in the use of using acoustic recognition techniques for avian population monitoring programs.

## Introduction

Recognition of conspecific individuals on the basis of vocalizations has been demonstrated for many bird species [1]. Acoustic identification of individuals can be used to evaluate territory boundaries, map home ranges, conduct population surveys, monitor population dynamics, and detect dispersal patterns within avian populations [2]. Unlike physical markers, which may affect the individuals' behavior [3], [4], survival rate, and reproduction [5], [6], individual recognition on the basis of vocalizations is non-invasive and low interference [7]. The use of vocalizations for ‘marking’ or identifying individuals in ornithological studies has increased in recent years [8], especially where the use of physical markers (e.g. colored rings) would be difficult to observe, where birds are extremely sensitive to disturbance, or where the capture and handling of birds poses many logistical or ethical issues [7].

Key to this being an effective, practical method of individual identification, acoustic recognition techniques must ensure that: 1) the vocalization used is easily recordable and the individual distinctive features persist over time; 2) the correct rate of acoustic recognition remain high even when sample sizes are large [7]. Vocal features can vary with social context [9], time of year [10], and body condition [11] and these changes may greatly obscure vocal individuality [12]. Previous studies have achieved a high correct rate of acoustic recognition in a number of avian species, demonstrating the temporal stability of vocal features over extended time-periods [13], [14]. Despite these encouraging findings, recordings from fewer than 30 individuals have been utilized in the majority of these studies [7], [15]. This raises the concern of whether the correct rate of acoustic recognition based on such low sample sizes is sufficiently robust, or whether similar correct rates can be achieved in studies that utilize larger avian populations (i.e. based on larger sample sizes of individual birds). If this were not to be the case, then this would greatly reduce confidence in the use of acoustic individual recognition as a suitable tool for studying individual avian behaviors and individual-based avian population monitoring studies.

In this paper we build on some of our previous acoustic recognition research [2] by collecting new huge data to examine the influence of sample size on the correct rate of acoustic recognition of individuality within a different, and larger color-banded population of Brownish-flanked Bush Warbler (*Cettia fortipes*). Males of this widespread and common south-east Asian species sing clear, high-pitched songs from shrubs throughout the breeding season [16], while females tend to remain silent all year. The songs of this species consist of an introductory whistled and a more complex syllable part. Based on the number of notes within the syllable part, two main song types can be identified: an alpha song type and a beta song type [17]. Most individuals sing both song types in alternation or rotation and consistently have only one variation within each song type. Other individuals sing only one type consisted of two variations within that type, or sing two song types and have two variations within one or both song types [2]. Song variations here are defined as the songs with different notes in the syllable portion within one song type, and can be classified visually using spectrograms owing to their consistent structure. Vocal features of this species exhibit little variation over the whole breeding season, and remain stable under different social contexts, such as spontaneous natural song, or songs induced by playback [2].

Providing data on the correct rate of bush warbler classification alone without any reference to the weighting of the variables that are used is insufficient to provide much insight on the features of the songs that are suitable for classification. Therefore we also provide a description of the intra- and inter-individual variation observed for the different measurements and identify the features of alpha and beta song types that are suitable for correct individual classification for Brownish-flanked Bush Warbler.

The highly complex and diverse nature of the alpha and beta types of this species enables us to measure a greater number of spectro-temporal variables. The second goal of this paper is to create an experimental simulation examining how the percentage of song types correctly classified may vary with increasing number of spectro-temporal variables incorporated into the analyses. Increasing the number of spectro-temporal variables measured may increase the resolution of distinguishing different individuals. Conversely, increasing spectro-temporal variables measured may be time-consuming and lead to difficulty with data independence issues when analytical techniques such as discriminant function analysis [18].

## Materials and Methods

### Bird Banding and Ethics Statement

Individual birds were trapped in mist nets using acoustic playback of the species' song. Once caught, the birds were immediately colour banded using a unique combination of 3 mm diameter plastic and metal rings by one of the authors (YZ) who is a senior bird-banding member of National Bird-banding Center of China. All birds were captured, ringed and released at the point of capture within eight minutes. This research protocol was approved by the Animal Care and Use Committee at the Beijing Normal University, the National Bird-banding Center of China (NBCC), under license number 201000042 and the Dongzhai National Nature Reserve under license number 2011002.

### Song Recording

Recordings of Brownish-flanked Bush Warbler songs were collected in Dongzhai National Nature Reserve (31°57′N, 114°15′E), south-central China. Warblers at the study sites inhabit artificial scrubland habitat dominated by tea plants (*Camellia sinensis*). Previous research has found that there are nearly 200 individuals that occupy territories within and around the periphery of the study area. Songs were recorded with a TASCAM HD-P2 portable digital recorder (Tascam Co., Japan) and a Sennheiser MKH416 P48 external directional microphone (Sennheiser Co., Germany). Songs were recorded at a sampling rate of 44.1 kHz and 16 bits. Recordings were primarily collected at distances of 1–10 m from May to June 2011. Recordings from 139 of these males were of sufficient high-quality to be incorporated into the subsequent analysis. Individuals were identified using plastic coloured bands. A previous study demonstrated the temporal stability over the whole breeding season (from May to August) of their vocal features [2], so repeat recordings were not deemed necessary. All the recordings were induced by playback.

### Song Measurement

We used Avisoft-SASLab Pro 4.3 (Avisoft Bioacoustics, germany; Specht 2005) to analyze songs. Using a band-pass filter, we removed noise below 1 kHz, which is below the frequency of Brownish-flanked Bush Warbler songs. For each male, we randomly selected and subsequently measured 10 songs per variations from our recordings. Concerning the alpha song type, we found 108 individual males sang only 1 variation; 24 individuals sang 2 variations; 1 individual sang 3 variations; with 6 other individuals not having an alpha song type. For the beta song type, we found that 111 individuals sang 1 variation; 8 individuals sang 2 variations; with 20 other individuals not having this song type. Overall, we measured a total of 1590 alpha songs and 1270 beta songs. For both song types we measured 1 spectro-temporal variable in the whistled component and 10 variables for each of the notes of the syllable component. Consequently, we measured 21 variables for the alpha song type and 31 for the beta song type. Details of the spectro-temporal variables are shown in Figure S1, Figure S2 and Table S1.

### Data Analysis

We compare the coefficients of variation within (CVw) and among (CVa) individual song variations. The Potential of Individual Coding (PIC) is the CVa/CVw ratio and indicates how great the amongst-variation was relative to the within-individual variation [19], [20]. We calculated the PIC for each variable as PIC = CVa/mean CVw [19], [20]. After confirming the PIC>1 which mean variability among song variations exceeds variability within song variations for most variables (Table 1 and 2), we used discriminate function analysis (DFA) to determine whether songs of different individuals could be distinguished from each other. Since most variations are in one-to-one correspondence with their related individuals within one song type, we use the variations as the independent sample for the DFA, whilst avoiding discrimination against song variations and individuals. We employ simulated methods to determine how accuracy of identifying individuality could be influenced by sample size (number of variations or individuals). This involved examining the correct rate of individual identification from randomly selected sub-sample sizes without replacement from our database. We selected initial sub-sample size of 20 variations and then further incremental increases in sub-sample sizes of 10 variations. Each sub-sample sizes were repeated (100 times) to randomly select.

**Table 1:** The details of coefficients of variation within (CVw), among (CVa) song variations, and PIC in alpha song.

Variables		CV within	CV among	PIC
whistled part	F3	0.01	0.12	14.91
1st note in syllable part	T1	0.06	0.18	3.08
	T2	0.12	0.15	1.26
	T3	0.44	0.36	0.8
	F1	0.04	0.19	5.26
	F2	0.08	0.27	3.2
	F3	0.11	0.12	1.09
	F4	0.09	0.31	3.34
	T4	0.02	0.13	5.49
	F5	0.47	1.67	3.53
	T5	0.02	0.11	6.27
2nd note in syllable part	T1	0.06	0.14	2.16
	T2	0.11	0.18	1.66
	T3	0.32	0.24	0.77
	F1	0.03	0.33	9.46
	F2	0.03	0.1	3.93
	F3	0.13	0.13	0.96
	F4	0.36	2.78	7.82
	T4	0.02	0.09	5.32
	F5	0.1	0.28	2.9
	T5	0.02	0.1	4.79

See Figure S2 and Table S1 for explanations of variables.

**Table 2:** The details of coefficients of variation within (CVw), among (CVa) song variations, and PIC in beta song.

Variables		CV within	CV among	PIC
whistled part	F3	0.01	0.11	14.43
1st note in syllable part	T1	0.08	0.17	2.18
	T2	0.11	0.14	1.29
	T3	0.42	0.31	0.73
	F1	0.03	0.25	8.58
	F2	0.05	0.18	3.80
	F3	0.10	0.12	1.19
	F4	0.17	1.17	6.85
	T4	0.02	0.11	5.83
	F5	0.23	0.66	2.86
	T5	0.01	0.10	7.66
2nd note in syllable part	T1	0.07	0.13	1.88
	T2	0.17	0.22	1.26
	T3	0.33	0.27	0.82
	F1	0.04	0.22	5.35
	F2	0.03	0.14	5.00
	F3	0.08	0.09	1.12
	F4	0.47	1.61	3.39
	T4	0.02	0.14	9.20
	F5	0.15	0.28	1.88
	T5	0.02	0.09	5.99
3rd note in syllable part	T1	0.09	0.17	1.90
	T2	0.20	0.25	1.26
	T3	0.40	0.33	0.81
	F1	0.03	0.11	3.29
	F2	0.01	0.12	10.49
	F3	0.04	0.08	2.32
	F4	0.27	0.53	1.98
	T4	0.02	0.07	2.93
	F5	0.76	2.13	2.82
	T5	0.01	0.08	7.97

See Figure S2 and Table S1 for explanations of variables.

The relationship between the correct rate of individual identification and number of spectro-temporal variables was examined using a similar DFA approach that involved incremental increases in the number of variables. First, we randomly selected 3 variables from our database and calculated the DFA correct rates. We then subsequently added random variables in increments of 3, each of which were repeated 100 times. Results from jack-knifed classifications are reported as percentages of songs correctly assigned. All statistical tests were conducted using R software (v.2.11.1; R Development Core Team 2011).

## Results

Variation among individuals for alpha song types was greater than within individuals, with mean CVw values ranging from 0.01 to 0.47, and mean CVa values ranged from 0.09 to 2.78. Similarly for beta song type, mean CVw values ranged from 0.01 to 0.76, and CVa values ranged from 0.07 to 2.13. The range of PIC values for alpha song type is 0.77 to 14.91 and beta song type 0.73 to 14.43. Except 6 variables concern the location of maximum amplitude within each note in syllable part, most variables are with PIC>1. Overall CVa values were greater than CVw values for 46 of 52 variables measured (Table 1 and 2).

**Figure 1:**
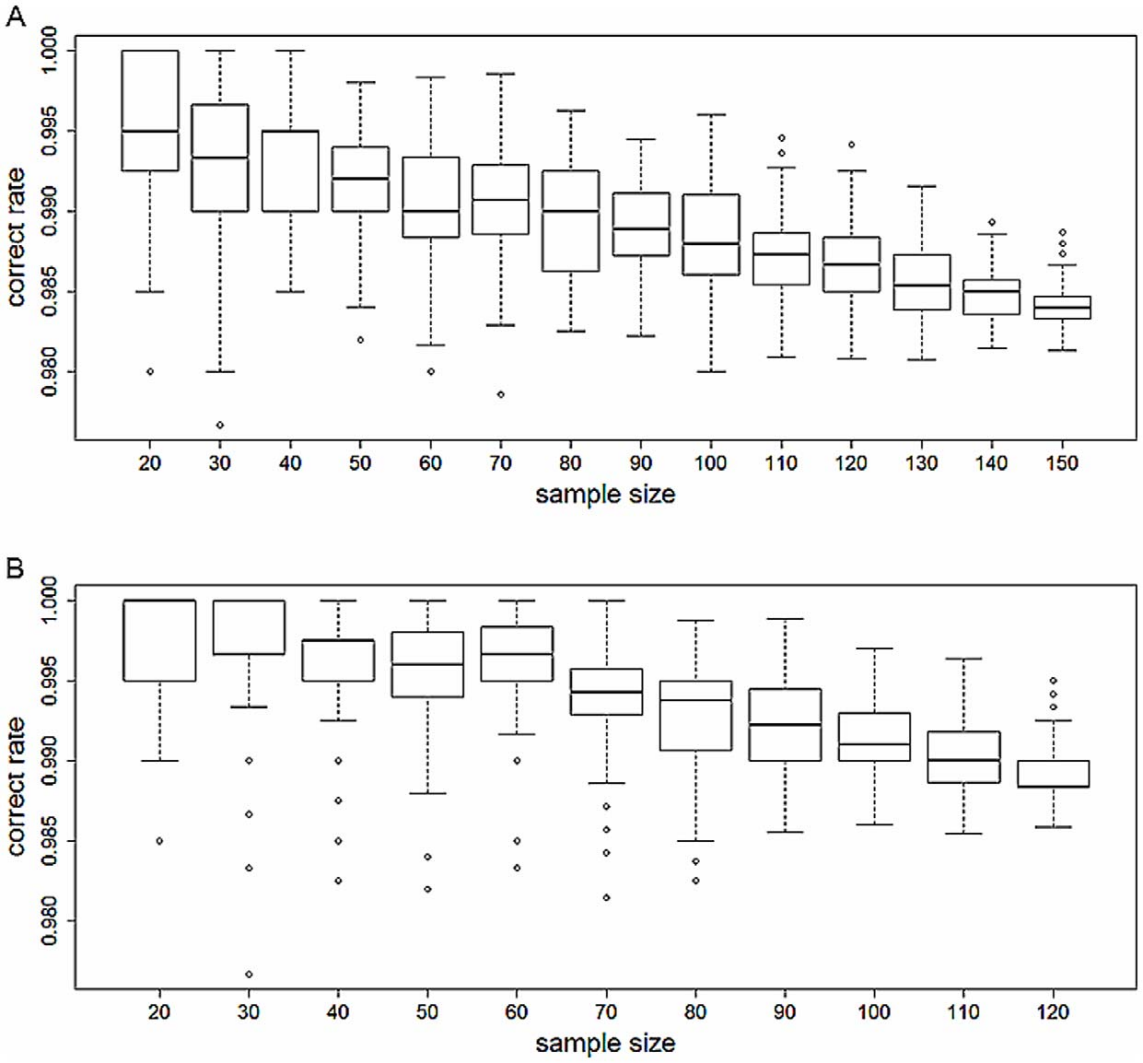
The correct rate of acoustic identification with different sample sizes for alpha (A) and beta(B) song type.

DFA yield a high correct rate of acoustic individual identification of individuals although there was a slight decline in the percentage correct rate of individual identification with increasing sample sizes for both alpha and beta song types (Fig. 1). For all the 139 individuals sampled, most songs were correctly classified regardless of whether alpha or beta song types were used. Of the alpha song types, 98.4% of all songs were correctly classified. Songs from 140 variations were 100% correctly classified, songs from 18 variations were 80–90% correctly classified, and only one variation was 50% correctly classified. For beta song type, 98.8% of all songs were correctly classified. The songs from 117 variations were 100% correctly classified, while songs from other 10 variations were 70%–90% correctly classified.

**Figure 2:**
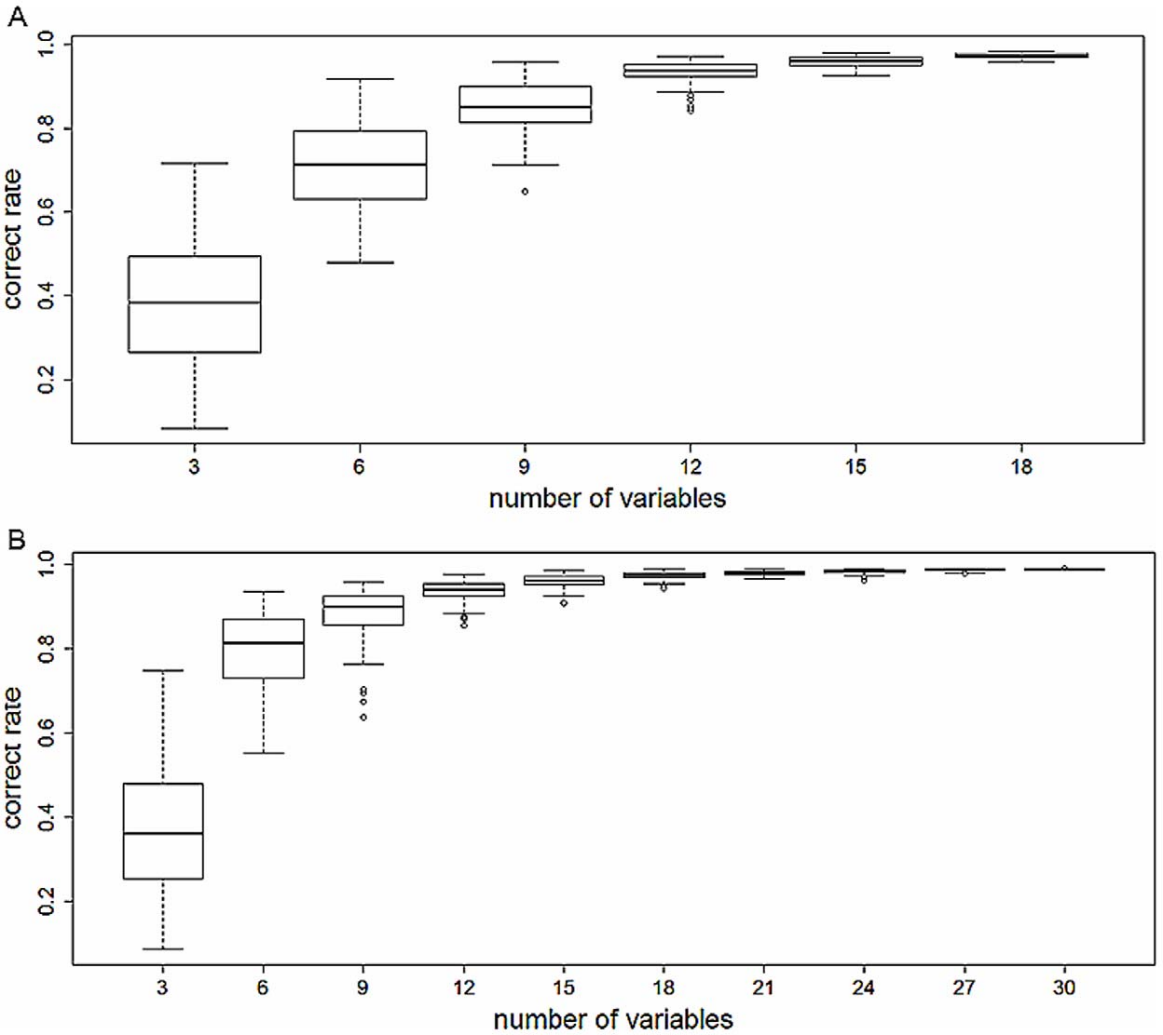
The correct rate of acoustic identification with different variable numbers using total sample size for alpha (A) and beta (B) song type.

The percentage of song types correctly classified increased with increasing number of variables incorporated into the analyses. DFA correct rate increased sharply with increasing the number of randomly assigned variables from 3 to 6, and continued to increase until reaching a stable value (with greatly reduced 95% confidence intervals) when more than 15 variables were included in the analysis (Fig. 2). When using only 3 or 6 variables, the DFA correct rate of individual identification was less than 80%. When the variable number increased to 12 of more variables, the mean DFA correct rates increased to greater than 90% (Fig. 2).

## Discussion

In this study we have demonstrated that it is possible to distinguish between large numbers of individual male Brownish-flanked Bush Warblers based only on the differences in their individual song structure. Previous studies have suggested that increasing the overall sample size of individual singing males would reduce the correct rate of individual identification. Holschuh (2004) suggested that because of greater overlap in vocal characteristics within neighbouring subsets of male Northern Saw-whet owls (*Aegolius acadicus*), reducing number of individuals for analyses from the larger population would greatly improved the correct rate of individual discrimination (http://web.unbc.ca/~otterk/publications/Holschuh%202004.pdf). In our study, most measured variables show greater variation in inter-individual than intra-individual (Table 1 and 2) and are suitable for acoustic identification. Six variables concern the location of maximum amplitude show PIC value less than 1. As the distance from target bird to the phone, and even the direction of bird head, may affect the song amplitude, so this variables show less individual feature may just due to the move of target individual during recording. Although we could not achieve the same correct rate of identification when comparing the correct rate achieved using total sample size of individuals and those with just a sub-sample, the correct rate (>98%) was still high (Fig. 1). Besides, we found that simply using an appropriate number of variables is sufficient to obtain a high correct rate - the mean DFA correct rates of identifying Brownish-flanked Bush Warbler was greater than 90% when only 12 randomly selected variables were used (Fig. 2). Both the slight decline in the percentage correct rate of individual identification with increasing sample sizes (Fig. 1), and the redundancy of variables after achieving a high correct rate (Fig. 2) imply that acoustic recognition is still an extremely useful technique even when population sample sizes may be larger than the 139 individuals sampled in our study of the Brownish-flanked Bush Warbler.

Most acoustic identification studies are based on samples sizes of fewer than 30 individuals [7], [15], which may represent only a small subsample of the true population size of many passerine species. Our study based on 139 of the *c.*200 individual total population size achieved a better correct rate compared with a previous study based on only 22 individuals of another Brownish-flanked Bush Warbler population situated *c.*1,000 km from our study area [2]. Other studies which focus on the practicality of using acoustic analysis to estimate avian population size have sampled >100 individuals [15], [21]. However these studies are based on the assumption that a high correct rate of acoustic identification from small samples is also indicative of larger populations without testing such an assumption, combine different location and acoustic data, and only clarify the song differences of several individuals from only one location.

To our knowledge, acoustic recognition at the individual level based on larger sample (population) sizes of more than 100 individuals under field conditions has not been undertaken for other avian species, and our work increases confidence of using acoustic recognition techniques for avian population monitoring programs.

## Supporting Information

Figure S1 **Sound spectrograms of Brownish-flanked Bush Warbler (** ***Cettia fortipes*** **) songs, show whistled part and terminal part both in alpha and beta song type.**
(TIF)

Figure S2 **Detailed illustration of measured spectro-temporal variables included in the DFA. Only variables measured from one note are present.**
(TIF)

Table S1 **Spectro-temporal variables illustrated in Figure S2.**
(DOC)
