# Supplementary figures and images for: Acoustic Identification of Individuals within Large Avian Populations: A Case Study of the Brownish-Flanked Bush Warbler, South-Central China
Source: PLoS One. 2012 Aug 6;7(8):e42528. doi: 10.1371/journal.pone.0042528 (PMC3412828; doi:10.1371/journal.pone.0042528)

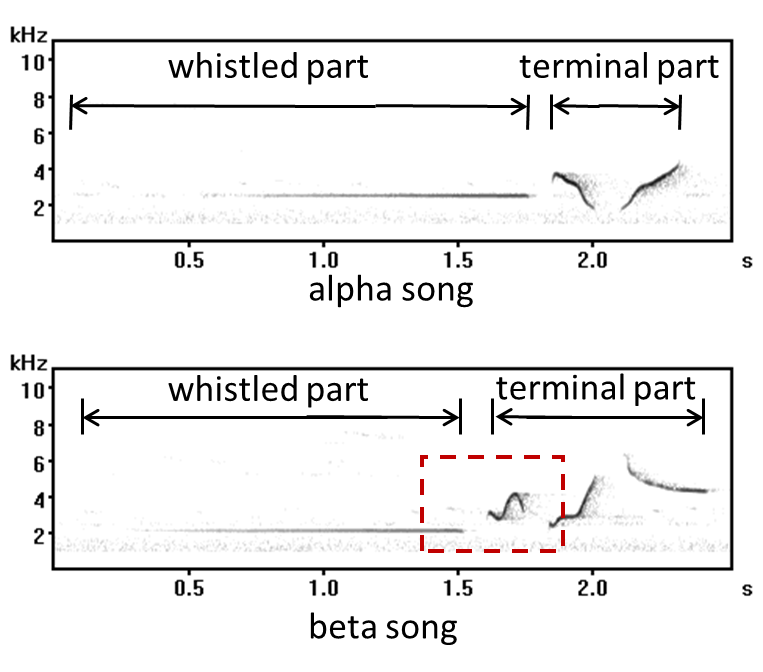

Supplement: Figure S1 — Sound spectrograms of Brownish-flanked Bush Warbler ( Cettia fortipes ) songs, show whistled part and terminal part both in alpha and beta song type. (TIF) [file pone.0042528.s001.tif]

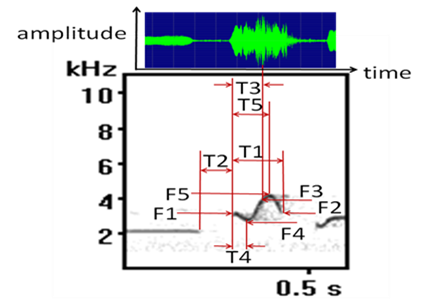

Supplement: Figure S2 — Detailed illustration of measured spectro-temporal variables included in the DFA. Only variables measured from one note are present. (TIF) [file pone.0042528.s002.tif]
