# Supplementary material for: Acoustic Identification of Individuals within Large Avian Populations: A Case Study of the Brownish-Flanked Bush Warbler, South-Central China
Source: PLoS One. 2012 Aug 6;7(8):e42528. doi: 10.1371/journal.pone.0042528 (PMC3412828; doi:10.1371/journal.pone.0042528)
Supplement: Table S1 — Spectro-temporal variables illustrated in Figure S2. (DOC) [file pone.0042528.s003.doc]

| Code | Spectro-temporal variable |
| --- | --- |
| T1 | Duration of note from start to end |
| T2 | Duration from the end of the preceding note to the start of the current note |
| T3 | Duration from the start of note to the location of the maximum amplitude within each note |
| F1 | Frequency at the start of each note |
| F2 | Frequency at the end of each note |
| F3* | Frequency of the maximum amplitude of the spectrum |
| F4 | Minimum frequencies of each note |
| T4 | Duration from the start of note to the location of the minimum frequency within each note |
| F5 | Maximum frequencies of each note |
| T5 | Duration from the start of note to the location of the maximum frequency within each note |

*only this variable was measured both in whistled part and notes within syllable part, while other variables were measured only in notes within syllable part.
